# Divergence of Catalytic Mechanism within a Glycosidase Family Provides Insight into Evolution of Carbohydrate Metabolism by Human Gut Flora

**DOI:** 10.1016/j.chembiol.2008.09.005

**Published:** 2008-10-20

**Authors:** Tracey M. Gloster, Johan P. Turkenburg, Jennifer R. Potts, Bernard Henrissat, Gideon J. Davies

**Affiliations:** 1 York Structural Biology Laboratory, Department of Chemistry, University of York, Heslington, York, YO10 5YW, UK; 2 Department of Biology, University of York, Heslington, York, YO10 5YW, UK; 3 Architecture et Fonction des Macromolécules Biologiques UMR6098 CNRS, Universités Aix-Marseille I & II, case 932, 163 Avenue de Luminy, 13288 Marseille cedex 9, France

**Keywords:** CHEMBIO, PROTEINS, MICROBIO

## Abstract

Enzymatic cleavage of the glycosidic bond yields products in which the anomeric configuration is either retained or inverted. Each mechanism reflects the dispositions of the enzyme functional groups; a facet of which is essentially conserved in 113 glycoside hydrolase (GH) families. We show that family GH97 has diverged significantly, as it contains both inverting and retaining α-glycosidases. This reflects evolution of the active center; a glutamate acts as a general base in inverting members, exemplified by *Bacteroides thetaiotaomicron* α-glucosidase *Bt*GH97a, whereas an aspartate likely acts as a nucleophile in retaining members. The structure of *Bt*GH97a and its complexes with inhibitors, coupled to kinetic analysis of active-site variants, reveals an unusual calcium ion dependence. ^1^H NMR analysis shows an inversion mechanism for *Bt*GH97a, whereas another GH97 enzyme from *B. thetaiotaomicron*, *Bt*GH97b, functions as a retaining α-galactosidase.

## Introduction

**Figure 1:**
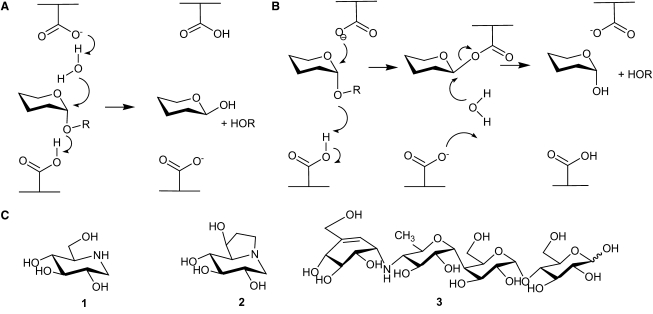
GH Mechanisms and α-Glucosidase Inhibitors (A and B) Hydrolysis with (A) inversion and (B) retention of anomeric configuration. (C) Inhibitors deoxynojirimycin (**1**), castanospermine (**2**), and acarbose (**3**).

Glycoside hydrolases (GHs), the enzymes responsible for cleavage of glycosidic bonds in mono-, di- and polysaccharides, are ubiquitous throughout all kingdoms of life, where they play myriad biological roles. GHs catalyze cleavage of the glycosidic bond with either net retention or inversion of anomeric configuration. The majority of the enzymes characterized to date tend to adopt the “classical” mechanisms seminally first proposed by Koshland (1953). Inversion of stereochemistry at the anomeric position involves a single-step mechanism (Figure 1A), in which protonation of the glycosidic oxygen by the general acid and leaving group departure is concomitant with nucleophilic attack of a water molecule that has been deprotonated by the general base. Typically, the general acid and base are enzymatic residues bearing a carboxylate group. Retention of stereochemistry involves a double displacement mechanism via a covalent intermediate (Figure 1B). During the first step, protonation of the glycosidic oxygen by the acid/base (acting as an acid) to aid leaving group departure is concomitant with nucleophilic attack by the nucleophile residue at the anomeric carbon, which leads to the formation of the covalent intermediate. Subsequently, the acid/base (acting as a base) deprotonates a nucleophilic water molecule (Zechel and Withers, 2000). A particularly rich source of glycosidases, within which one can study the evolution of carbohydrate active enzyme systems and their mechanisms, is the bacterium *Bacteroides thetaiotaomicron*. Analysis of the *B. thetaiotaomicron* genome has demonstrated that the organism possesses a significant number of genes (6.6% of the entire genome) that encode carbohydrate-processing enzymes, including many GHs; this is the largest proportion of genome devoted to carbohydrate metabolism of any organism sequenced to date (Davies et al., 2005).

*B*. *thetaiotaomicron*, a Gram-negative anaerobe, is a symbiotic component of the microflora in the human gut; the flora perform a range of beneficial metabolic tasks that are not encoded by our own genome (Comstock and Coyne, 2003; Xu et al., 2003; Zocco et al., 2007). *B. thetaiotaomicron* plays a major role in the breakdown of polysaccharides ingested in the diet into a form that could otherwise not be absorbed and utilized by the host (Comstock and Coyne, 2003; Xu et al., 2003; Zocco et al., 2007). Perhaps the most well-characterized polysaccharide utilization machinery in *B. thetaiotaomicron* is that encoded by the *sus* operon. The *sus* operon contains seven genes (*susA*–*susG*) that encode proteins involved in the utilization and metabolism of starch (D'Elia and Salyers, 1996; Reeves et al., 1997), and is regulated by the activator SusR in response to high levels of glucose oligomers (Cho et al., 2001). *susA* encodes a neopullanase, which hydrolyzes the α-1,4 linkages in starch to produce smaller oligosaccharides; *susB* encodes an α-glucosidase, which is able to break down these smaller oligomers into glucose; *susC*–*susF* encode outer membrane proteins involved in starch binding; and *susG* encodes a protein, the role of which is unclear, but which has high sequence similarity to amylases (D'Elia and Salyers, 1996; Reeves et al., 1997).

Carbohydrate active enzymes have been classified into families based upon their amino acid sequence similarities (Coutinho and Henrissat, 1999). Currently there are 113 sequence-distinct glycosidase families. A feature of almost all classical CAZy families is that, since sequence dictates structure, and structure determines function, the catalytic mechanism is conserved within a sequence-based family (Henrissat and Davies, 1997). Exceptions to this rule are rare and unusual: GH4 and GH109 enzymes are not classical hydrolases, but instead use NAD^+^ in a transient reduction/oxidation reaction with leaving group elimination (Rajan et al., 2004), and GH23 is a family with both inverting and retaining hexosaminidases, but the catalytic mechanism of neither is understood, and may involve substrate participation in catalysis. The α-glucosidase encoded by the *susB* gene belongs to family GH97 (Hughes et al., 2003) of this Carbohydrate Active enZymes (CAZy) classification (http://www.cazy.org/), the catalytic mechanism of which was unknown prior to this work, but had been predicted to be retaining in an insightful bioinformatics analysis (Naumoff, 2005). *B. thetaiotaomicron* possesses a total of 10 GH97 members, and there are currently 69 other bacterial GH97 open reading frames, and one from the archaea *Haloarcula marismortui*. The goal of our work was to probe the structural and functional diversity within this family in order to shed light on the evolution and diversity of carbohydrate metabolism in the human gut.

Here we describe the three-dimensional structure and mechanistic characterization of the enzyme encoded by *susB*, a GH97 α-glucosidase from *B. thetaiotaomicron* (*Bt*GH97a). The native structure, together with three-dimensional complexes with the inhibitors deoxynojirimycin (**1**) and castanospermine (**2**), in addition to kinetics of wild-type and mutant enzymes, reveals an unusual metal ion-dependent catalysis. Structural similarity searches confirm the earlier sequence predictions (Naumoff, 2005) that the enzyme is related to family GH27 retaining α-galactosidases (see Fujimoto et al., 2003; Garman et al., 2002). Close inspection of the structural overlap, however, revealed no obvious candidate nucleophile, and ^1^H NMR analysis showed that *Bt*GH97a is an inverting enzyme, consistent with the observed disposition of catalytic groups and in contrast to earlier predictions made *in silico*. Examination of GH97 sequences, in light of the structure, suggests an early evolutionary divergence into two main subfamilies, in only one of which the catalytic constellation presented by *Bt*GH97a is conserved. The second subfamily is shown to be a subfamily of retaining enzymes, demonstrated here for the *B. thetaiotaomicron* α-galactosidase *Bt*GH97b by ^1^H NMR analysis. Family GH97 shows a clear evolutionary divergence into both inverting and retaining enzymes, which now, by virtue of the three-dimensional structure and chemical analysis, allows prediction of catalytic mechanism for other family members.

## Results

Family GH97 comprises around 80 members at present, 10 of which are from *B. thetaiotaomicron*. Two members, SusB (hereafter, *Bt*GH97a) from *B. thetaiotaomicron* (Smith and Salyers, 1991) and an enzyme from *Tannerella forsythensis* (Hughes et al., 2003) have been investigated previously, and both have been shown to possess α-glucosidase activity. *Bt*GH97a was shown to hydrolyze a range of substrate sizes from maltose to maltoheptaose, and both α-1,4 and α-1,6 linkages (Smith and Salyers, 1991).

### *Bt*GH97a Three-Dimensional Structure and Activity

**Figure 2:**
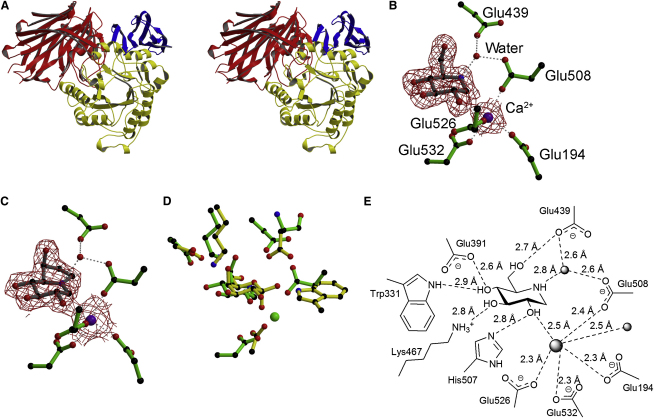
Structural Insights into *Bt*GH97a (A) Divergent stereo ribbon representation of *Bt*GH97a; the N-terminal domain is in red, the core (β/α)_8_ domain in yellow, and the C-terminal domain in blue. (B and C) Ball-and-stick representation of *Bt*GH97a in complex with (B) **1** and (C) **2**; observed electron density for the maximum likelihood weighted 2*F*_obs_ − *F*_calc_ map is contoured at 1σ. The purple spheres represent calcium ions. (D) Ball-and-stick representation of the active-site overlap between *Bt*GH97a and a GH27 enzyme (PDB ID code 1UAS). Figures were drawn using BOBSCRIPT (Esnouf, 1997). (E) Interactions between *Bt*GH97 and **1**.

The structure of *Bt*GH97a was solved using X-ray crystallography data to 1.90 Å resolution (Table 1). Phasing was performed using single-wavelength anomalous dispersion data collected at the peak wavelength (0.9795 Å) for selenium, and the majority of the protein was built automatically. The tertiary structure of *Bt*GH97a reveals three domains (Figure 2A); an N-terminal β-super-sandwich domain (residues 1–317), followed by a canonical (β/α)_8_ barrel (residues 318–636) and a C-terminal β sheet domain (residues 637–738). A calcium ion is present in the (β/α)_8_ barrel domain; it is octahedrally coordinated by four glutamate residues (Glu194, Glu508, Glu526, and Glu532) and both oxygen atoms of a molecule of ethylene glycol, which was sequestered from the cryoprotectant. The calcium ions in each molecule of the asymmetric unit in the native structure (and the complexes described below) yield atomic B factors of 14–16 Å^2^ when refined with unitary occupancy. Superposition with other (β/α)_8_ barrel GHs (data not shown), as well as the presence of the four glutamates, suggests that the calcium ion is, in fact, bound in the active-site region of the enzyme. We initially assumed that it would be inhibitory, as is often seen when cations bind in place of the cationic transition state (Brzozowski et al., 2000; Tailford et al., 2007).

The calcium ion displays high apparent affinity and may only partially be removed using EDTA or Chelex. Partial removal of the calcium causes a substantial reduction in activity, but the enzyme maintains a residual activity, confirming high metal ion affinity. Other divalent ions, including magnesium and zinc, caused inactivation of the enzyme. The addition of calcium to the EDTA-treated enzyme resulted in restoration of full activity; all further kinetics experiments were thus conducted in the presence of 10 mM CaCl_2_, at which the enzyme had full activity.

The pH dependence of k_cat_/K_M_ for *Bt*GH97a was fitted to a bell-shaped ionization curve (see Supplemental Data available online), which gave p*K*_a_ values of 5.5 and 7.7; all subsequent kinetics were performed at the enzyme optimum, pH 6.6. Kinetic parameters for *Bt*GH97a were determined with the aryl α-glycosides dinitrophenyl α-d-glucopyranoside (DNP-Glc) and *p*-nitrophenyl α-d-glucopyranoside (*p*NP-Glc), and with the α-1,4 linked disaccharide maltose, Table 2. The k_cat_/K_M_ was highest for the most activated substrate, DNP-Glc (∼2000 s^−1^ mM^−1^), followed by *p*NP-Glc (620 s^−1^ mM^−1^), but only modest for maltose (0.0072 s^−1^ mM^−1^). The lower activity on maltose may reflect the enzyme's preference for longer substrates (Smith and Salyers, 1991).

### Probing *Bt*GH97a through Inhibition

The inhibition of *Bt*GH97a using classical α-glycosidase inhibitors yielded K_i_ values of 59 μM for deoxynojirimycin **1**, 59 μM for castanospermine **2**, and 108 nM for acarbose **3**. Isofagomine, calystegine B_2_, and isofagomine lactam did not inhibit the enzyme at 100 μM. The structures of *Bt*GH97a in complex with **1** and **2** were determined at 1.7 and 2.0 Å resolution, respectively. Clear 2*F*_obs_ − *F*_calc_ electron density in the active site (of both molecules of the asymmetric unit) allowed facile building of the complexes (Figures 2B and 2C). Both **1** and **2** are found in a slightly flattened (toward ^4^*E*) ^4^*C*_1_ chair conformation. Each of the inhibitors and the surrounding active-site residues superpose perfectly, so only the interactions for *Bt*GH97a in complex with **1** are described (Figure 2E). Hydrogen bonds are formed between the hydroxyl group at C6 and Glu439, between the hydroxyl at C4 and Glu391 and Trp331, and between the hydroxyl at C3 and Lys467. The hydroxyl group at the C2 position interacts with the calcium ion and with His507. The ring nitrogen atom of **1** interacts with a water molecule, which is also within hydrogen bonding distance of Glu439 and Glu508, and hence may mimic the catalytic water molecule in an inverting mechanism (described below in light of ^1^H NMR experiments). The calcium ion assumes an octahedral coordination, with six ligands, as was observed in the native structure. It coordinates the same four glutamate residues (Glu194, Glu508, Glu526, and Glu532), but instead of interacting with an ethylene glycol molecule, it now coordinates the hydroxyl group at C2 of **1** and a water molecule (all at a distance of between 2.3 and 2.5 Å; see Supplemental Data).

### Dissection of *Bt*GH97a Mechanism Using Active-Site Variants

Each of the glutamate residues in the unusual active-site constellation of *Bt*GH97a (Glu194, Glu439, Glu508, Glu526, and Glu532) were mutated in turn to alanine. Where activity allowed, full Michaelis-Menten kinetics were performed using *p*NP-Glc as a substrate. Additionally, substrate depletion methods, using DNP-Glc as a substrate, were used to determine the k_cat_/K_M_ directly (Table 2). Mutations of Glu194 and Glu532 allowed measurable activity with *p*NP-Glc; in both cases, the K_M_ was higher and the k_cat_ lower than for the wild-type enzyme. Overall the k_cat_/K_M_ was reduced around 70-fold for E194A and 80-fold for E532A. Activity using DNP-Glc as a substrate could be measured for all mutants except E508A. The k_cat_/K_M_ was reduced around 7-fold for E194A, 250,000-fold for E439A, 40-fold for E526A, and 4-fold for E532A. These values are discussed below in light of further mechanistic investigations.

### GH97 Structural and Sequence Alignments

As predicted in an insightful bioinformatics analysis (Naumoff, 2005), searches for the nearest structural homologs to *Bt*GH97a using SSM (Krissinel and Henrick, 2004) revealed the closest matches were to GHs from family 36 (an α-galactosidase from *Thermotoga maritima*; PDB ID code, 1ZY9 [unpublished]) and family 27 (including an α-galactosidase from rice; PDB ID code, 1UAS [Fujimoto et al., 2003]). Although *Bt*GH97a shares low sequence similarity with the sequences from GH27 and GH36, *Bt*GH97a superposes with a root-mean-square deviation between Cα atoms of 3.3 Å over 391 residues with the closest GH36 structure (1ZY9) and 3.3 Å over 277 residues with the closest GH27 structure (1UAS). GH27 and GH36 both belong to clan GH-D in the CAZy classification (Coutinho and Henrissat, 1999), and are known to perform catalysis with net retention of anomeric configuration (Comfort et al., 2007).

A global overlap of *Bt*GH97a with the GH27 α-galactosidase homolog from rice, for which there is a ligand complex (PDB ID code, 1UAS), reveals partial conservation of the active-site residues (Figure 2D). The galactose present in the GH27 structure overlays well with **1** bound in the active site of *Bt*GH97a. Asp391, Lys467, and Glu532 from *Bt*GH97a superpose well with Asp51, Lys128, and Asp185 (the catalytic acid/base), respectively, from the GH27 enzyme. There are no reports of a metal ion bound in the active site of the GH27 or GH36 enzymes, and, although Glu532 appears in a similar position to the acid/base residue from the GH27 enzyme, the other three glutamate residues that coordinate the calcium ion are not conserved. There is also a large difference in the region surrounding the catalytic nucleophile (Asp130) in the GH27 enzyme, where, in the equivalent position in *Bt*GH97a was, to our surprise, a glycine residue (Gly469).

This surprising lack of a candidate catalytic nucleophile for *Bt*GH97a made the prediction of the stereochemical outcome unclear. The distance between the two key catalytic residues in a three-dimensional structure (if identified) are often useful in suggesting the mechanism adopted by the enzyme; in the case of retaining enzymes, the distance between the two residues is, on average, 5.5 Å, whereas it is often larger for inverting enzymes, as both the substrate and water need to fit into the active site simultaneously (Zechel and Withers, 2000). However, the number of glutamate residues in the active site of *Bt*GH97a makes it difficult to predict the mechanism for this family with certainty; hence, the stereochemistry was determined unambiguously by NMR.

### Determination of *Bt*GH97a Mechanism

**Figure 3:**
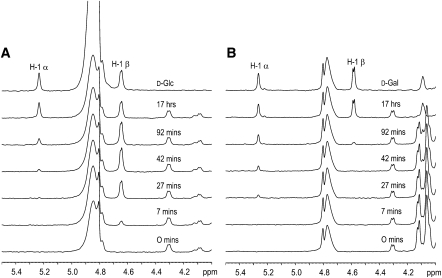
^1^H NMR Analysis of Stereochemical Outcome (A and B) Hydrolysis using (A) *Bt*GH97a with *p*NP-Glc and (B) *Bt*GH97b with *p*NP-Gal. The times indicate the beginning of the spectra acquisition, and peaks for the α- and β-anomers are shown (assigned according to Roslund et al. [2008]).

The stereochemistry of *Bt*GH97a hydrolysis using ^1^H NMR was determined by following the product of the enzymatic reaction and its subsequent mutarotation to the thermodynamically favored ratio of α- and β-anomers. *Bt*GH97a hydrolysis of *p*NP-Glc was monitored over 17 hr (Figure 3A). Only the β-anomer of glucose was observed to accumulate initially; as mutarotation occurred, however, the amount of the β-anomer began to decline and the α-anomer appeared, until the standard equilibrium of around 64% β-anomer/ 36% α-anomer was reached. The data unequivocally demonstrate that the reaction hydrolyzed by *Bt*GH97a proceeds with inversion of stereochemistry at the anomeric position to generate the β-d-glycoside as product. The three-dimensional structure had already suggested the likely candidate for the *Bt*GH97a catalytic base as Glu439. Glu439 sits on the “β-face,” and, in the complexes with **1** and **2**, it coordinates a water molecule in a position for in-line attack at the anomeric center (Figure 2E). In addition, the E439A mutant was 250,000-fold less efficient at hydrolyzing DNP-Glc than the wild-type enzyme, which is consistent with loss of base catalytic assistance to water attack. The comparison with the GH27 retaining enzymes shows that Glu439 is ∼1.6 Å “further away” from the anomeric center compared with the aspartate nucleophile (Asp130) in the GH27 enzyme. Thus, this simple shift in the position of the carboxylate provides the extra space required for a single-step inverting mechanism.

Assignment of the catalytic acid is more troublesome, given the constellation of glutamates in the active site. Glu532 is isostructural with the known acid/base residue in the GH27 enzyme, suggesting that it may act as the catalytic acid. Furthermore, the E532A mutant had a far greater effect on *p*NP-Glc hydrolysis (80-fold) compared with the 4-fold change on DNP-Glc; given the greatly reduced need for protonic assistance with the latter substrate in these kinetics, and in light of the overlap with the GH27 enzyme, there is compelling evidence for the role of Glu532 as the catalytic acid.

### Relating Sequence to Mechanism in GH97

**Figure 4:**
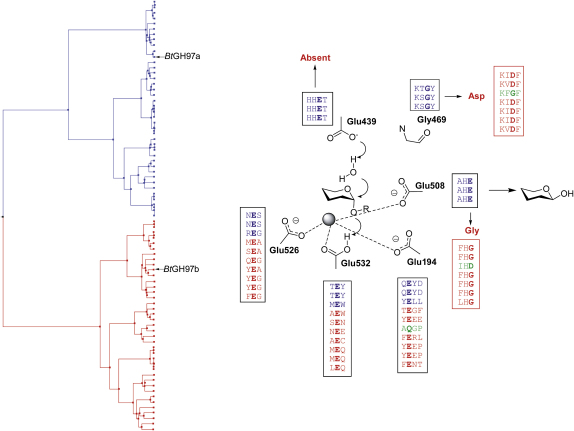
GH97 Family Tree, Active Center Geometry, and Sequence Alignments for the Key Active-Site Residues of the *B. thetaiotaomicron* Representatives Inverting subfamily sequences are shown in blue and retaining subfamily sequences in red. The inverting-retaining transition reflects a loss of the inverting base and its replacement by a Gly-Asp shift elsewhere in the sequence. GH97 has six unusual sequences, which appear to contain neither inverting nor retaining catalytic signatures; the *B. thetaiotaomicron* outlier is shown in green. The full annotated family tree is given in Supplemental Data.

Sequence alignments of the whole GH97 family gave rather intriguing results when the positions of the five active-site glutamate residues were examined. The residues equivalent to Glu526 and Glu532 in *Bt*GH97a (both coordinate the calcium ion, and Glu532 is predicted to be the catalytic acid) were absolutely conserved throughout all members. The residue equivalent to Glu194, which is also important in coordinating the calcium ion, is also highly conserved, implying that calcium dependence may be universal in this family. Intriguingly, comparison of the residues equivalent to Glu439 in *Bt*GH97a, predicted to be the catalytic base, shows that, in around half of the family members, this is in a conserved HHET motif; however, in the other members, there is a highly variable sequence in this region. Given our proposal that Glu439 is the key catalytic base in the inversion mechanism, and it is almost essential for catalysis in *Bt*GH97a, this observation suggests a different catalytic mechanism for some of the other family members. Furthermore, the vast majority of these other GH97 members, which lack the putative base, have their own signature motif, (K/M)(I/V)DF, with the aspartate lying in exactly the same position as the nucleophilic aspartate (Asp130 in the PDB ID code 1UAS) of their GH27 relatives. A phylogenetic tree derived from the multiple-sequence alignment of 79 family GH97 members corroborates this finding with the clear definition of two subfamilies. In the *Bt*GH97a-like members (which possess a putative base for inversion), a glycine (Gly469 in *Bt*GH97a) in the motif K(T/S)GY is found where the aspartate residue is found in the second subfamily (Figure 4). Analysis of the whole family thus suggests that around half of the GH97 members possess a catalytic base, and would likely act with inversion by virtue of identity to *Bt*GH97a, but that an Gly-Asp change in the other half of the family would generate, by analogy to GH27, a catalytic constellation consistent with retention via a covalent glycosyl-enzyme intermediate.

### Mechanistic Studies on a Second GH97 Enzyme

To ascertain whether an enzyme that possessed the “opposite” set of motifs to *Bt*GH97a did in fact proceed with retention of configuration, as sequence alignments would suggest, we cloned and overexpressed another open reading frame encoding a GH97 enzyme from *B. thetaiotaomicron* (*Bt*GH97b). This enzyme, however, did not hydrolyze DNP-Glc or *p*NP-Glc, but was shown to be active on *p*-nitrophenyl α-d-galactopyranoside (*p*NP-Gal), revealing that it is an α-galactosidase. It did, however, display an identical metal ion dependency profile to *Bt*GH97a. The pH dependence of k_cat_/K_M_ for *Bt*GH97b was more difficult to ascertain than for *Bt*GH97a, as activity levels appeared to change very little with pH values between 4 and 8; kinetics were therefore performed at pH 6.6 (the optimum for *Bt*GH97a). Although it is often dangerous to interpret pH rate profiles (Knowles, 1976), the flattening here may be related to the unusual presence of a catalytic calcium ion, which will impact on the p*K*_a_ of the acid/base and nucleophile and any potential Lewis-acid assistance to leaving group departure. Kinetic parameters for *Bt*GH97b were determined with the aryl α-glycoside *p*NP-Gal and the α-1,6 disaccharide melibiose (Table 2). The catalytic efficiency, reflected by the k_cat_/K_M_, was higher for *p*NP-Glc (391 s^−1^ mM^−1^) than for melibiose (0.011 s^−1^ mM^−1^).

*Bt*GH97b hydrolysis of *p*NP-Gal was monitored over 17 hr by ^1^H NMR (Figure 3B). During the first hour, the α-anomer of galactose accumulated, with no evidence of any β-anomer, demonstrating that α-galactose was the initial product from the α-galactoside substrate, and that the enzyme indeed acted with retention of anomeric configuration.

## Discussion

GH97 enzymes can be classified into two broad subfamilies of inverting and retaining enzymes (Figure 4; see also the Supplemental Data), breaking the dogma that stereochemistry is conserved within a classical GH family; this also contradicts previous bioinformatics studies, which predicted a retaining mechanism for hydrolysis (Naumoff, 2005). Our studies also demonstrate an expansion in the substrate specificity previously determined for GH97 enzymes, as previously only α-glucosidase activity had been reported (Hughes et al., 2003; Smith and Salyers, 1991). As only two were tested here, and *B. thetaiotaomicron* alone possesses 10 GH97 enzymes, it suggests that there may be further substrate diversity to discover within this fascinating family. Each of the enzymes was shown to be extremely specific for either glucose- (*Bt*GH97a) or galactose- (*Bt*GH97b) containing substrates, demonstrating absolute selectivity at the C4 position. Furthermore, GH97 enzymes appear to possess strong metal ion dependence, which is rare among GHs. GH2 *Escherichia coli* β-galactosidase (*lacZ*) is magnesium ion-dependent (Juers et al., 2001; Richard et al., 1996; Selwood and Sinnott, 1990; Sinnott and Withers, 1978), and family 38 and 47 α-mannosidases are zinc and calcium ion dependent, respectively. These α-mannosidases are observed to coordinate a metal ion to the hydroxyl groups at the C2 and C3 positions of inhibitors, which is thought to play a role in substrate specificity and/or distortion (Karaveg et al., 2005; Vallée et al., 2000; van den Elsen et al., 2001). The calcium ion in GH97 enzymes, however, may provide acid assistance to the catalytic acid during hydrolysis, as has been suggested for the magnesium ion in the *E. coli* β-galactosidase. The presence of interactions between the calcium ion and four glutamate residues in *Bt*GH97a, as well as the hydroxyl group at C2 of the inhibitors, suggests that the enzyme has evolved a way to keep the ion bound tightly and that it is vital to function.

The power of sequence alignments, in conjunction with the structure of *Bt*GH97a and experimental demonstration of reaction stereochemistry, has demonstrated beautifully how the consensus motifs surrounding a few key residues can be used to predict the mechanism for hydrolysis for the majority of the GH97 enzymes. Our analysis, however, indicates there are six sequence “outliers” which fall into the part of the tree containing the enzymes which proceed with retention, but where the sequences appear not to contain the consensus motifs consistent with retaining or inverting enzymes; one of these sequences is from *B. thetaiotaomicron*, as highlighted in green in Figure 4. At the position equivalent to Gly469 in *Bt*GH97a, inverting enzymes possess the motif K(T/S)GY, whereas retaining enzymes contain (K/M)(I/V)DF. The six outliers possess the motif KXG(Y/F), which implies that they lack the catalytic nucleophile. Similarly, at the position equivalent to Glu508 in *Bt*GH97a, inverting enzymes have a conserved HE motif, whereas retaining enzymes have HG; in the six exceptions, one possesses an HE motif, while the other five sequences have an HD motif. Examination of the residues equivalent to Glu439 in *Bt*GH97a shows that the inverting enzymes possess an absolutely conserved HHET, whereas the sequence is apparently random for the retaining enzymes at this position. Strangely, however, of the six exceptions, although one possesses an HNET motif, the rest have a variable sequence, suggesting that they also lack the residue equivalent to the catalytic base for the inverting mechanism; hence, they appear to lack both the base and the enzyme-derived nucleophile. In addition, while the glutamate equivalent to Glu194 in *Bt*GH97a is a highly conserved glutamate residue in both inverting and retaining enzymes, the six outlier sequences all possess a glutamine residue at this position. These intriguing observations suggest that these members may either be inactive or that they have evolved yet another catalytic apparatus, adding to the remarkable plasticity of family GH97. Inactive glycosidase variants that have evolved to perform other activities have been described in other families, most notably GH18 (Durand et al., 2005) and GH13 (Bröer and Wagner, 2002; Janeček et al., 1997), and bioinformatics analysis has predicted that the same may be true of GH57 (Zona et al., 2004).

Simple repositioning of a base or nucleophile has been reported as a route to “man-made” changes in reaction stereochemistry, notably by Wang et al. (1994) and Kuroki et al. (1995). These authors insightfully foresaw a route to mechanistic change that only now has been shown to have evolved in nature by these GH97 enzymes. One further case in which two families, related by sequence and structure, showed different stereochemistries was reported by Alberto et al. (2004); the positioning of the catalytic apparatus, however, is invariant, and the change in mechanism appears to be achieved by subtle repositioning of the sugar itself.

GH97 is thus an extremely rare family of GHs in which enzymes have unambiguously been shown to proceed with both inversion and retention of stereochemistry. In light of the three-dimensional structure and in conjunction with superpositions with other clan members, careful dissection of nuances in the sequence alignments has aided this discovery. In all other families, with the exception of GH4 (which are NAD^+^ dependent and not classical hydrolases) and GH23 (where the basis for retention is not understood), the mechanism is conserved. The metal dependence for the GH97 enzymes indicates a special feature that possibly helps to create an environment that supports both the retaining and inverting mechanisms. Evolutionary pressure must have conferred an advantage to similar enzymes with the ability to utilize mechanisms with different stereochemical outcomes. It is only as we begin to unlock these subtleties and secrets that nature has imposed during evolution that we can begin to predict enzyme specificity and mechanism with any certainty in the future.

## Significance

**Cleavage of the glycosidic bond, catalyzed by glycoside hydrolases (GHs), yields one of two stereochemical outcomes in which configuration of the reactive anomeric center is either retained or inverted. These two reactions follow one of two canonical mechanisms featuring double displacement via a covalent intermediate, or a single nucleophilic displacement by water, respectively. The difference in mechanism reflects the dispositions of the important functional groups within the active site of the enzyme, which is essentially conserved within the 113 sequence-based GH families. Our studies demonstrate that family GH97 enzymes have diverged significantly from this general model, as it contains both inverting and retaining α-glycosidases. This divergence of mechanism reflects a comparatively small change in the active center, in which a glutamate plays the role as a general base in inverting members, whereas, in retaining members, an aspartate residue from a different place in the sequence likely acts as the nucleophile. The three-dimensional structure and kinetic analyses of *Bt*GH97a reveal an unusual calcium ion-dependent catalytic center in which a glutamate residue is poised to activate the nucleophilic water molecule with both general acid and calcium ion assistance to leaving group departure. Bioinformatics analysis of the whole sequence family reveals an evolutionary split between inverting and retaining family members, reflecting subtle changes to the active-center environment, which is possibly aided by the metal ion. In addition, these sequence alignments reveal an intriguing six sequences that appear to possess neither the base for inversion nor the nucleophile for retention of configuration. Evolutionary pressures must have encouraged these subtle differences between closely related enzymes to confer a biological advantage on those able to hydrolyze substrates to both α- and β-anomer products. It is only as we dissect these variations imposed by nature that mechanisms and specificity can be predicted with certainty in the future.**

## Experimental Procedures

### Gene Cloning, Protein Production, and Protein Purification

The genes encoding *Bt*GH97a and *Bt*GH97b (GenBank accession numbers AAO78808 and AAO76978, respectively) were amplified using the polymerase chain reaction from *B. thetaiotaomicron* VPI-5482 genomic DNA using primers that gave ligation-independent cloning (LIC) compatible ends. These were ligated into an LIC-modified pET28a vector using standard procedures (Bonsor et al., 2006). Protein production and purification was identical for each protein. Plasmid containing the gene of interest was transformed into *E. coli* BL21 (DE3) cells, and cultured in 0.5 L autoinduction media (Studier, 2005) supplemented with 50 μg ml^−1^ kanamycin at 37°C for 8 hr. Protein production was induced at 30°C overnight. Cells were harvested and resuspended in 20 mM HEPES, pH 7, 150 mM NaCl, and lysed by sonication. The supernatant was applied to a 5 ml HisTrap nickel-Sepharose column (GE Healthcare), preequilibrated in the same buffer, and the protein eluted from an imidazole gradient. The protein was dialyzed to remove the imidazole, concentrated, and purified further on an S200 16/60 gel filtration column, preequilibrated in 20 mM HEPES, pH 7, 150 mM NaCl. Selenomethionine-containing *Bt*GH97a was obtained and purified in the same way as described for the native protein, except that the autoinduction media contained selenomethionine (SeMet).

### Crystallization

*Bt*GH97a, at 10 mg ml^−1^, was crystallized from 18%–22% polyethylene glycol 3350 and 0.02 M sodium/potassium phosphate. Crystals were cryoprotected in a solution containing the relevant mother liquor with the addition of 25% ethylene glycol, and were flash frozen in liquid nitrogen. Crystals were also grown in the presence of 2.5 mM **1** or **2** using the same crystallization conditions.

### Data Collection, Structure Solution, and Model Refinement

Data for both the native and the SeMet *Bt*GH97a were collected from single crystals at 100K on beamline ID23-1 at the European Synchrotron Radiation Facility (ESRF), Grenoble, France. Data were integrated and scaled using HKL2000 (Otwinowski and Minor, 1997), and all computing used the CCP4 suite of programs (CCP4, 1994), unless otherwise stated. Phasing from SeMet residues was performed in SOLVE (Terwilliger and Berendzen, 1999), and initial model building was carried out with RESOLVE (Terwilliger, 2003). The output model from RESOLVE was entered as a starting model in ARP/wARP (Lamzin and Wilson, 1993), which was used to trace the majority of the protein chain. Subsequent refinement involved alternate rounds of model building in COOT (Emsley and Cowtan, 2004) and likelihood-based refinement in REFMAC (Murshudov et al., 1997). Subsequent to our structure determination, the structure of native SusB was also released (PDB ID code 2D73) by M. Kitamura, M. Okuyama, Y. Kitago, F. Tanzawa, H. Mori, A. Kimura, M. Yao, and I. Tanaka. Data for *Bt*GH97a in complex with **1** and **2** were also collected on beamline ID23-1 at the ESRF, and were handled in the same way as described for the native data.

### Site-Directed Mutagenesis of *Bt*GH97a

Mutations (E194A, E439A, E508A, E526A, and E532A) were introduced into the gene encoding *Bt*GH97a using a Quikchange site-directed mutagenesis kit (Stratagene) by the standard protocol described in the kit manual. Resulting plasmids were sequenced (Applied Biosystems 3130 sequencer) to check that the desired mutation had been incorporated. Mutant proteins were produced and purified using the same protocol as described for the native protein.

### Enzyme Kinetics

Methods for the enzyme kinetics are described in full in the Supplemental Data. Briefly, kinetic studies involving a spectrophotometric product were conducted by monitoring the change in UV/visible absorbance with a Cintra 10 spectrophotometer. All experiments were carried out at 37°C, in a total volume of 1 ml and in the presence of 10 mM CaCl_2_. Enzyme dependence on pH, ranging from pH 4 to 8, was carried out using substrate depletion methods to determine the k_cat_/K_M_ directly with *p*NP-Glc for *Bt*GH97a or *p*NP-Gal for *Bt*GH97b as the substrate. *p-*Nitrophenolate release was monitored continuously at 400 nm and the data fitted to a first-order rate equation using GRAFIT (Leatherbarrow, 2001). k_cat_/K_M_ values at different pH values were fitted to a bell-shaped ionization curve to determine the p*K*_a_ values of the ionizable groups. Individual kinetic parameters were determined at the pH optimum for catalysis using DNP-Glc or *p*NP-Glc for *Bt*GH97a and the E194A and E532 mutants, and *p*NP-Gal for *Bt*GH97b. 2,4-Dintrophenolate/*p*-nitrophenolate release was monitored at 400 nm and the rates determined. Kinetic parameters were determined by fitting the data to the Michaelis-Menten equation in GRAFIT. Substrate depletion methods were used to determine the k_cat_/K_M_ values for each of the five mutants with DNP-Glc. K_i_ values for **1**–**3** with *Bt*GH97a were determined under steady-state conditions at low substrate concentration using *p*NP-Glc. Activity was monitored at 400 nm for 300 s, and the rates determined. The fractional decrease of the rate in the absence and presence of inhibitor was used to determine the K_i_, which was taken as the mean of triplicate readings.

Kinetics were also performed using a stopped assay, where glucose was detected using glucose oxidase/peroxidase linking enzymes (Megazyme). Measurements were made with maltose as the substrate for *Bt*GH97a and melibiose for *Bt*GH97b. Aliquots were taken at time intervals over 10 min, boiled to inactivate the enzyme, and 1 ml of the glucose oxidase/peroxidase solution was added. Assays were otherwise performed according to the manufacturer's instructions. Reaction rates were determined and the data fitted to the Michaelis-Menten equation in GRAFIT. Substrate inhibition was observed with *Bt*GH97a and fitted appropriately.

### NMR

^1^H NMR spectra were acquired on a Bruker 700 MHz spectrometer at 25°C, with presaturation for water suppression. Samples had been lyophilized and diluted in 100% ^2^H_2_O twice prior to the experiment. Substrates *p*NP-Glc for *Bt*GH97a and *p*NP-Gal for *Bt*GH97b were present at a final concentration of 0.5 mM. Assays were run in 10 mM maleate buffer, pH 6.6, 10 mM CaCl_2_, with the addition of 75 μM trimethylsilylpropionic acid as an internal chemical shift reference. Substrate hydrolysis was initiated by the addition of enzyme (in the same buffer) at a final concentration of approximately 15 nM. Spectra (with 16 scans, 16,384 data points, and a 16.5 s relaxation delay between scans) were collected at regular intervals for 17 hr, at which time hydrolysis appeared to be complete. Control spectra of the substrate in the absence of enzyme, and of 0.5 mM glucose and 0.5 mM galactose in 100% ^2^H_2_O, were also acquired.

### Bioinformatics

Sequences were retrieved from the National Center for Biotechnology Information (http://www.ncbi.nlm.nih.gov) from their accession number found in CAZy family GH97 (http://www.cazy.org/fam/GH97.html). The amino acid sequences were aligned using MUSCLE (Edgar, 2004). The aligned sequences were clustered using the SECATOR algorithm (Wicker et al., 2001), which relies on BIONJ (Gascuel, 1997) to build a tree from the multiple-sequence alignment, and subsequently collapses the branches from subtrees after identification of the nodes joining different subtrees (Wicker et al., 2001). The neighbor-joining tree was made from the resulting distance matrix using Blosum62 substitution parameters. Visualization of the tree was accomplished with an in-house program.

## Figures and Tables

**Table 1:** Data Processing and Refinement Statistics for *Bt*GH97a

Data Collection^a^	*Bt*GH97a (SeMet)	*Bt*GH97a (Native)	*Bt*GH97a in Complex with **1**	*Bt*GH97a in Complex with **2**
Resolution (Å)	15−2.50 (2.59−2.50)	15−1.90 (1.97−1.90)	15−1.66 (1.72−1.66)	20−2.00 (2.07−2.00)
Space group	*P*2_1_	*P*2_1_	*P*2_1_	*P*2_1_
Unit cell parameters				
*a*, *b*, *c*; β	75.6, 111.9, 102.5; 100.8°	75.6, 111.6, 102.4; 100.9°	75.7, 112.2, 102.5; 100.9°	74.6, 112.0, 104.1; 100.9°
R_merge_	0.084 (0.16)	0.084 (0.28)	0.075 (0.35)	0.076 (0.35)
Mean I/σI	15.9 (8.6)	8.9 (2.3)	20.0 (3.4)	13.3 (2.2)
Completeness (%)	100 (99)	91 (57)	99 (93)	95 (69)
Multiplicity	5.3 (5.0)	2.3 (1.7)	5.4 (3.3)	3.5 (2.5)
No. unique reflections	57,815	120,776	195,947	108,971
R_cryst_	—	0.15	0.15	0.17
R_free_	—	0.20	0.19	0.23
Rmsd bonds (Å)	—	0.014	0.014	0.015
Rmsd angles (°)	—	1.41	1.41	1.50
Rmsd chiral volume (Å^3^)	—	0.103	0.109	0.105
PDB ID code	—	2JKA	2JKE	2JKP

Values in parentheses represent outer shell. Rmsd, root-mean-square deviation.

a Beamline (ESRF) ID23-1.

**Table 2:** Kinetic Data for *Bt*GH97a with DNP-Glc, *p*NP-Glc and Maltose, *Bt*GH97b with *p*NP-Gal and Melibiose, and *Bt*GH97a Mutants with *p*NP-Glc and DNP-Glc

Enzyme/Mutant	Substrate	K_M_ (mM)	k_cat_ (s^−1^)	k_cat_/K_M_ (s^−1^ mM^−1^)
*Bt*GH97a	DNP-Glc	0.14 ± 0.01	280 ± 7	1997
	*p*NP-Glc	0.085 ± 0.009	53 ± 2	620
	Maltose^a^	2.0 ± 0.4	0.015 ± 0.002	0.0072
*Bt*GH97b	*p*NP-Gal	0.23 ± 0.01	91 ± 2	391
	Melibiose	1.5 ± 0.17	0.015 ± 0.0004	0.011
*Bt*GH97a				
E194A	*p*NP-Glc	0.31 ± 0.02	2.8 ± 0.07	9.2
E532A	*p*NP-Glc	0.14 ± 0.02	1.1 ± 0.04	7.7
Wild-type	DNP-Glc	—	—	1807 ± 68
E194A	DNP-Glc	—	—	255 ± 3
E439A	DNP-Glc	—	—	0.007 ± 0.001
E508A	DNP-Glc	—	—	ND
E526A	DNP-Glc	—	—	48 ± 4
E532A	DNP-Glc	—	—	402 ± 29

Kinetic data for *Bt*GH97a with DNP-Glc, *p*NP-Glc and maltose, *Bt*GH97b with *p*NP-Gal and melibiose, and *Bt*GH97a mutants with *p*NP-Glc were determined using full Michaelis-Menten kinetics, and those for *Bt*GH97a mutants with DNP-Glc were determined using substrate depletion methods. Where no data are reported for *Bt*GH97a mutants with *p*NP-Glc, no activity could be measured. ND, none detected.

a Substrate inhibition was observed. The K_i_ value for maltose was 17.2 mM.

## Accession Numbers

Coordinates and structure factors have been deposited in the Protein Databank with accession codes 2JKA, 2JKE, and 2JKP.

## Supplemental Data

Document S1. Supplemental Experimental Procedures and Three Figures
