# Supplementary material for: Divergence of Catalytic Mechanism within a Glycosidase Family Provides Insight into Evolution of Carbohydrate Metabolism by Human Gut Flora
Source: Chem Biol. 2008 Oct 20;15(10-3):1058–67. doi: 10.1016/j.chembiol.2008.09.005 (PMC2670981; doi:10.1016/j.chembiol.2008.09.005)
Supplement: Document S1. Supplemental Experimental Procedures and Three Figures [file mmc1.pdf]

Chemistry & Biology *15*

Supplemental Data

Divergence of Catalytic Mechanism within a Glycosidase Family Provides Insight into Evolution of Carbohydrate Metabolism by Human Gut Flora

Tracey M. Gloster, Johan P. Turkenburg, Jennifer R. Potts, Bernard Henrissat, and Gideon J. Davies

Supplemental Experimental Procedures

Enzyme kinetics

Kinetic studies involving a spectrophotometric product were conducted by monitoring the change in UV/visible absorbance with a Cintra 10 spectrophotometer, equipped with a Thermocell Peltier power supply. All experiments were carried out at 37 °C, in a total volume of 1 mL.

Enzyme dependence on pH was carried out using substrate depletion methods to determine the k_{cat}/K_M directly. Assays were run in 100 mM buffer (citrate buffer from pH 4 to 6, maleate buffer from pH 6 to 7 and HEPES from pH 7 to 8), 10 mM $CaCl_2$, 1 mg mL^{-1} bovine serum albumin (BSA) (except below pH 5 where it precipitated), and either 10 µM *p*-nitrophenyl α-D-glucopyranoside (*p*NP-Glc) for *Bt*GH97a or 15 µM *p*-nitrophenyl α-D-galactopyranoside (*p*NP-Gal) for *Bt*GH97b. The reaction was initiated by the addition of 10 µL enzyme to a final concentration of between 10 and 640 nM for *Bt*GH97a or 20 and 40 nM for *Bt*GH97b. *p*-Nitrophenolate release was monitored continuously at 400 nm for 10 min. Data (absorbance at 400 nm against time) were fitted to a first order rate equation ($A_t = A_0$ (1-exp(-kt))+offset, where A_t is the absorbance at time t, A_0 is the absorbance at time 0 and k is the rate constant) using GRAFIT to give V_{max}/K_M, which was adjusted for the enzyme concentration to give k_{cat}/K_M. The k_{cat}/K_M values at different pH values were fitted to a bell-shaped ionisation curve (k_{cat}/K_M = (limit x $10^{(pH-pKa1)}/(10^{(2xpH-pKa-pKa2)} + 10^{(pH-pKa1)}+1)$ to determine the pK_a values of the ionisable groups.

Enzyme activity was measured using between 0.02 and 0.8 mM dinitrophenyl α-D-glucopyranoside (DNP-Glc), 0.02 and 1 mM *p*NP-Glc for *Bt*GH97a and 0.02 and 1.2 mM *p*NP-Gal for *Bt*GH97b, in 100 mM maleate buffer, pH 6.6 (the optimum for enzyme catalysis) with the same additional components described above. The reaction was initiated with the addition of 10 µL enzyme to a final concentration of 2 nM for *Bt*GH97a with DNP-Glc and 10 nM for *Bt*GH97a with *p*NP-Glc and *Bt*GH97b with *p*NP-Gal. 2,4-dintrophenolate/*p*-nitrophenolate release was monitored continuously at 400 nm (the molar extinction coefficient was determined by measuring the absorbance of an accurately determined concentration of the substrate which had been hydrolysed to completion with a highly concentrated stock of enzyme) for 300 s, and the rates were taken as the slope between 100 and 200 s. The data were fitted to the Michaelis Menten equation in GRAFIT, which was used to calculate the K_M and V_{max}; the V_{max} could be adjusted for the enzyme concentration to give the k_{cat}.

Kinetics on the E194A and E532A mutants were conducted in the same way to determine the k_{cat} and K_M values. E194A, at a final concentration of 100 nM, was assayed at *p*NP-Glc concentrations between 0.05 and 1.5 mM and E532A, at a final concentration of 200 nM, was assayed at *p*NP-Glc concentrations between 0.05 and 1 mM. In addition, substrate depletion methods were used to determine the k_{cat}/K_M values for each mutant with

10 μM DNP-Glc; wild type was present at a final concentration of 10 nM, E194A at 140 nM, E439A at 1.5 μM, E526A at 260 nM and E532A at 150 nM. No activity could be determined for E508A using either substrate.

K_i values for **1-3** with *Bt*GH97a were determined under steady state conditions at low substrate concentration. Assays contained 25 μM *p*NP-Glc, 100 mM maleate buffer, pH 6.6, 1 mg mL^{-1} BSA and 10 mM $CaCl_2$. Concentrations of **1** ranged from 15 to 200 μM, **2** ranged from 10 to 150 μM and **3** ranged from 25 nM to 1 μM; *Bt*GH97a was present at a final concentration of 2 nM. Reactions were initiated by the addition of substrate following a 20 minute pre-incubation of the enzyme with inhibitor to prevent any complications from slow onset inhibition. Rates were monitored for a 300 s period, and the rates were taken as the slope of the line between 200 and 300 s. The fractional decrease of the rate in the absence and presence of inhibitor was used to determine the K_i using the equation $v_0/v_i = 1 + [I]/K_i$ (where v_0 is the rate in the absence of inhibitor, v_i is the rate in the presence of inhibitor, and [I] is the inhibitor concentration) and the mean K_i value was taken.

Kinetics were also performed using a stopped assay, whereas glucose was detected using glucosose oxidase/peroxidase linking enzymes (Megazyme). Assays were performed at 37 °C in 100 mM sodium maleate buffer, pH 6.6, 1 mg mL^{-1} BSA and 10 mM $CaCl_2$. Measurements were made at maltose concentrations between 0.25 and 8 mM for *Bt*GH97a and melibiose concentrations between 0.25 and 16 mM for *Bt*GH97b. 200 μL aliquots were taken at regular time intervals over the course of 10 min, boiled for 2 min to inactivate the enzyme, and 1 mL of the glucose oxidase/peroxidase solution was added. Assays were otherwise performed as in the manufacturer's instructions. Reaction rates were determined and the data fitted to the Michaelis Menten equation in GRAFIT; substrate inhibition was observed with *Bt*GH97a, and a better fit was obtained by fitting to the equation $v = (V_{max}[S])/(K_M + [S] + ([S]^2/K_i))$ (where v is the rate of the reaction, [S] is the substrate concentration and K_i is the inhibition constant for the substrate).

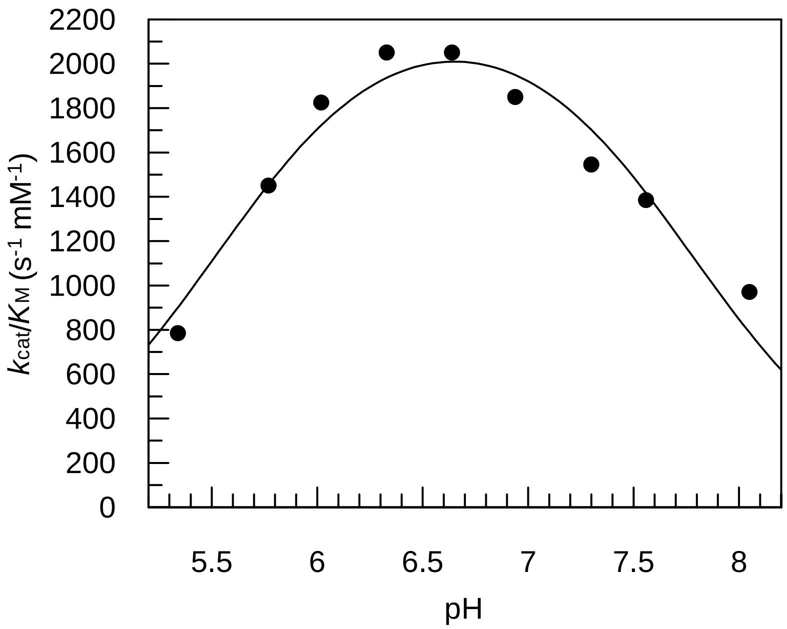

Figure S1. pH Dependence of k_{cat}/K_M for *Bt*GH97a

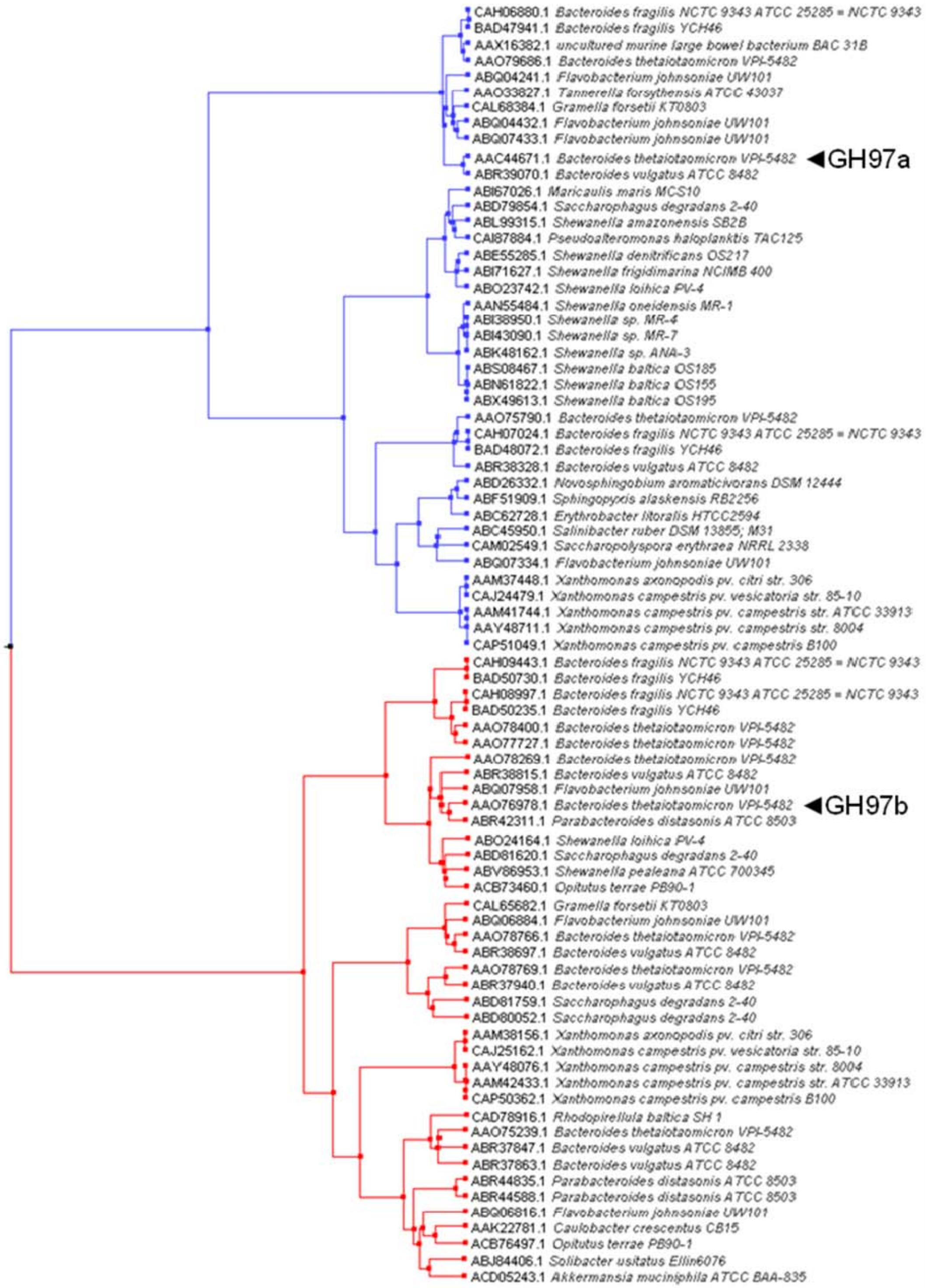

Figure S2. Family Tree Analysis of GH97 Members
*Bt*GH97a and *Bt*GH97b are highlighted, which represent the inverting enzymes in blue and the retaining enzymes in red.

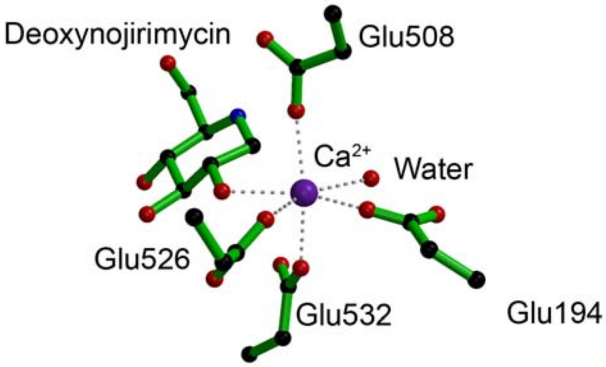

Figure S3. Calcium Coordination in the Active Site of *Bt*GH97a in Complex with Deoxynojirimycin
